# Change in Mechanical Properties of Laser Powder Bed Fused AlSi7Mg Alloy during Long-Term Exposure at Warm Operating Temperatures

**DOI:** 10.3390/ma16247639

**Published:** 2023-12-14

**Authors:** Emanuela Cerri, Emanuele Ghio

**Affiliations:** Department of Engineering and Architecture, University of Parma, Via G. Usberti, 181/A, 43124 Parma, Italy; emanuela.cerri@unipr.it

**Keywords:** additive manufacturing, long-term heat treatment, aging heat treatment, mechanical properties, microstructure, heterogeneous microstructure

## Abstract

Al–Si–Mg alloys are most commonly used to produce parts by laser powder bed fusion for several industrial applications. A lot of papers have already focused on the effects induced by conventional heat treatments on the microstructure and mechanical properties of AlSi10Mg alloys, rather than on AlSi7Mg. Nobody has investigated thermal stability during long-term direct and artificial aging heat treatments of AlSi7Mg. This study investigates the changes in mechanical properties induced by long-term exposure (512 h) at 150 and 175 °C (the operating temperature of AlSi7Mg) after (i) the laser powder bed fusion process performed on a pre-heated build platform (150 °C), and (ii) heat treatments to the solution at 505 °C per 0.5 and 4 h. Thermal stability was evaluated through both Vickers microhardness measurements to obtain the aging profiles, and tensile tests to evaluate the mechanical properties in specific conditions. An optical microscope was used to investigate the microstructure. It was found that aging at 175 °C confers the same effects induced by a secondary aging heat treatment on as-built samples and, simultaneously, the worst effects on the solution heat treated AlSi7Mg alloy after long-term exposure. The AlSi7Mg DA at both 150 °C and 175 °C showed the same Vickers microhardness (~95 HV0.5), UTS (~300 MPa), and YS (~200 MPa) values for the longest exposure times because the fine and cellular α-Al matrix confers higher stiffness and strength despite the over-aged conditions. On the other hand, the coarsening effects that affected the precipitates during aging at 175 °C, as well as the formation of the precipitate-free zones along the grain boundaries, justified the highest detrimental effects induced on the SHTed samples.

## 1. Introduction

Additive manufacturing is a recent production technique that has revolutionized the aerospace, automotive, biomedical, and construction industries. It can manufacture complex-shaped components by reducing both the cost and production time [1]. In this scenario, laser powder bed fusion (L-PBF) is one of the most suitable processes to additively manufacture metal components, especially the near-eutectic and age-hardenable AlSi7Mg. The aluminum alloys based on the Al–Si–Mg ternary system occupy a large portion of published studies because they satisfy several manufacturing qualifications and project requirements. In the former case, the short solidification range $\left(∆T=\sim 40\;K\right)$ and the low solidification shrinkage make these alloys suitable for the L-PBF process in which a laser source selectively scans and melts several successively deposited layers of powder. In the latter case, their high specific strength, wear resistance, and corrosion resistance meet the restrictive requirements imposed by several applications in the previously mentioned industrial fields [2,3].

The non-equilibrium conditions reached during the solidification process promote the formation of fine α-Al cells surrounded by nano-sized Si-eutectic particles that are disposed of in a network structure [2,4,5]. Fast cooling rates also lead to a silicon-supersaturated α-Al solid solution, thus restricting the precipitation of nano-sized Si particles and the Mg_2_Si phase [2,6]. Several other studies [2,7,8], however, have recorded an increment in the precipitation phenomena with the gain in temperature of the build platform where the samples were additively manufactured. In this scenario, a literature review accepted the following precipitation path [9,10]:

SSS → β″ → β′ → β-Mg_2_Si

where the β″ and β′ are the pioneering precipitates of the incoherent β-Mg_2_Si phase. Li et al. [11] added the pre-β″ term due to its precipitation during the aging at 150 °C of a cast Al–Si–Mg alloy. Zhu et al. [8], instead, highlighted the presence of nano-sized Si-particles and β″ phases in the α-Al cells through TEM observations. These microstructural features led the yield strength (YS) values to an increment of about 9% in AlSi10Mg alloy L-PBFed on a build platform pre-heated at 150 °C. When the build platform was heated to 200 °C, both the α-Al grains and Si-eutectic particles in the eutectic network were affected by coarsening phenomena [12,13]. Simultaneously, faster kinetics of the precipitation phenomena promote a decrease in strength [12,14]. On the other hand, as investigated in our previous studies [5,15,16], the mechanical properties of both AlSi7Mg and AlSi10Mg components can be considered height-dependent. The zones closest to the build platform showed higher mechanical properties than the farthest ones due to the different exposure times at the build platform temperature.

Due to the age-hardenability of Al–Si–Mg alloys, as well as the effects induced by several process parameters, research has investigated heat treatments to improve mechanical properties. Most papers focus on the effects induced by different heat treatments (T5 and T6) on the as-built AlSi10Mg alloys [6,7,8,12,17,18,19,20,21,22,23,24,25,26,27,28,29,30,31], rather than those on AlSi7Mg [4,32,33,34,35].

T5 heat treatment, which is a direct aging (DA), is generally performed in a temperature range between 150 and 250 °C and for exposure times that vary in relation to (i) the chemical composition of aluminum alloys (wt% of Si and Mg atoms) and (ii) the as-built conditions (e.g., build platform temperature) [5,9,15,16,26]. If the precipitation hardening were considered the unique phenomenon characterizing the as-built samples, then DA should be performed at temperatures lower than 200 °C [5,9]. Coarsening phenomena of both precipitates and Si-eutectic particles, the dissolution of the Si-eutectic network and grain growth affect the as-built microstructure at DA temperatures above 200 °C [2,9,13]. In this context, the highest Vickers microhardness, yield and ultimate tensile strength (UTS) are obviously reached in peak aged samples characterized by an undamaged Si-eutectic network.

Traditional T6 heat treatment, that mainly characterizes the as cast Al–Si–Mg alloys, is carried out with a solution heat treatment (SHT) temperature in the range between 400 °C and the eutectic temperature, for exposure times of 1–10 h, and with consecutive artificial aging (AA) that follow the DA conditions previously exposed [2]. During SHT, the fine α-Al cells and the Si-eutectic network are completely replaced by a composite-like structure where the Si-eutectic particles are embedded in a coarsened or recrystallized α-Al matrix [2,9,15,32]. The subsequent precipitation hardening (AA) recovers the mechanical properties that were reduced during the SHT [2,15,16,26,28]. Unfortunately, the exposure at SHT temperature decreases the sample density due to the expansion of the trapped gas and the consequent increase in the size of the porosities [30,31,36].

Research articles focused on heat treated L-PBF AlSi10Mg alloy occupy 90% of studies published since 2020 [37]. Few studies [27,32,38,39] have investigated the effects of long-term heat treatments carried out on L-PBF AlSi10Mg. On the other hand, no published studies describe the effects of long-term DA and T6 heat treatments induced on L-PBF AlSi7Mg samples that are characterized by mechanical properties’ variation along their height. For these reasons, and considering that the thermal stability of metallic components is very important in engineering applications, the present study aims to investigate the changes in mechanical properties during long-term exposure (up to 512 h) for DA and AA at 150 and 175 °C. These two temperatures cover the typical operating temperature range in which AlSi7Mg and AlSi10Mg alloys work [40]. Lastly, two different exposure times (0.5 and 4 h) at the SHT temperatures were considered to evaluate the effects on both the mechanical properties and microstructural heterogeneity. Furthermore, the Vickers microhardness measurements are discussed in relation to the Hollomon–Jaffe parameter (HJP) to remove the dependence from the various temperatures and time used during the DA and AA heat treatments.

## 2. Materials and Methods

### 2.1. Material and L-PBF Process

The AlSi7Mg samples were additively manufactured using a SLM^®^280 machine (Nikon SLM solution, Lübeck, Germany) under the processing conditions previously reported in [16]. A duration of about 70 h characterized the entire manufacturing process. Table 1 lists the chemical composition of the gas-atomized powders used for the L-PBF process.

**Figure 1:**
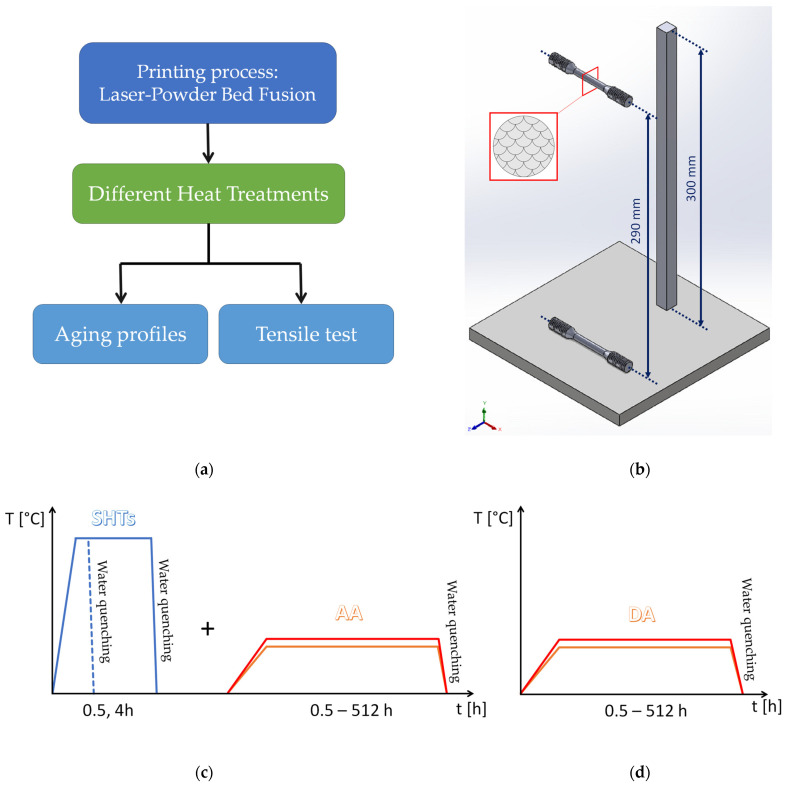
(**a**) A description of the research; (**b**) schematical representation of the bars and tensile samples analyzed in several heat treatment conditions (**c**,**d**). The performed heat treatments are: (**c**) SHT at 505 °C × 0.5, 4 h followed by AA at 150 °C and 175 °C per 0.5–512 h, (**d**) DA at 150 °C and 175 °C per 0.5–512 h.

Figure 1a shows the brief procedure of the present investigation. Several bars and billets were laser powder bed-fused on a build platform pre-heated at 150 °C, as schematically illustrated in Figure 1b. Horizontally arranged cylindrical tensile samples, fabricated up to a height of 300 mm, had a gauge length of 30 mm and a diameter of (6.0 ± 0.1) mm, according to the ASTM E8/E8M standard [41]. To evaluate the effect induced by the analyzed heat treatment conditions (Figure 1c,d), both the bars and tensile samples were divided into two groups depending on the fabrication distance from the platform: the ones at the bottom (at 0–15 mm from the build platform) and the ones at the top (at 285–300 mm from the build platform). This subdivision was driven by our previous studies [5,15,16] in which the microstructural features and mechanical properties of laser powder bed-fused AlSi7Mg, in as-built and peak aged conditions, have already been studied. The same studies will be considered in the present research to introduce the as-built and peak aged microstructural features.

For a better understanding of the results, the bottom regions were soaked at 150 °C (build platform temperature) for about 70 h, while the top ones were soaked for ~2 h due to both the total height of the bars and the duration of the manufacturing process.

### 2.2. Heat Treatments

The heat treatments (Figure 1c,d) used in the present study were the DA and SHT + AA. In the former case, the direct aging heat treatments were carried out at (i) 150 °C, which would continue to promote the effects of the pre-heated build platform, and (ii) at 175 °C for an exposure time between 0.5 and 512 h (Table 2 and Figure 1d). These temperatures also approximate well the operating temperature in which the AlSi7Mg alloy can work. In the latter case, the same aging conditions were employed on both samples solubilized at 505 °C per 0.5 and 4 h (Table 2 and Figure 1d). Heat treated samples were water-quenched after both the SHT and the aging. The exposure time and temperatures were chosen in relation to our previous studies and considering the aims of this study [15,16], to better explain the mechanical properties’ evolution during the long-term heat treatments: DA at 175 °C for an exposure time lower than 16 h, and SHT at 505 °C + AA at 175 °C per soaking time lower than 32 h.

Due to the precipitation gradient mentioned above, the DA was performed on samples taken from the top and bottom regions of the as-built bars, while the AA samples were heat treated regardless of their initial position (because of the effects induced by the SHT on the as-built microstructure).

### 2.3. Mechanical Testing

Microhardness was measured on AlSi7Mg samples, according to conditions listed in Table 2. Each value was the average of nine indentations [16]. The Vickers microhardness tests were executed by a VMTH Leica (Leica, Germany, Wetzlar) tester by using a load of 500 gf and a dwell time of 15 s.

Tensile cylindrical samples (Figure 1b) were tested in the as-built, solubilized, and aged conditions. In the latter case, two different conditions were chosen: (i) the peak aging, and (ii) the time and temperature at which the microhardness values exponentially decrease, according to the aging curves. Tensile tests were conducted at room temperature by using a servo-hydraulic machine (Z100, Zwick/Roell, Ulm, Germany) with a strain rate of 0.008 s^−1^.

### 2.4. Microstructural Chatacterization

The microstructure of both the DA and AA samples was observed through an inverted optical microscope (DMi8, Leica, Wetzlar, Germany) by cutting the bulk perpendicularly to the build direction. The sample surface was first mechanically ground and polished with silica colloidal suspension, and then chemically etched with Keller’s reagent (2.5 mL HNO_3_ + 1.5 mL HCl + 1 mL HF + 95 mL distilled water). A microstructural investigation of the as-built features was conducted by using a SEM (Scanning Electron Microscope Auriga, Zeiss, Oberkochen, Germany).

## 3. Results

### 3.1. Microstructure in Heat Treated Conditions

**Figure 2:**
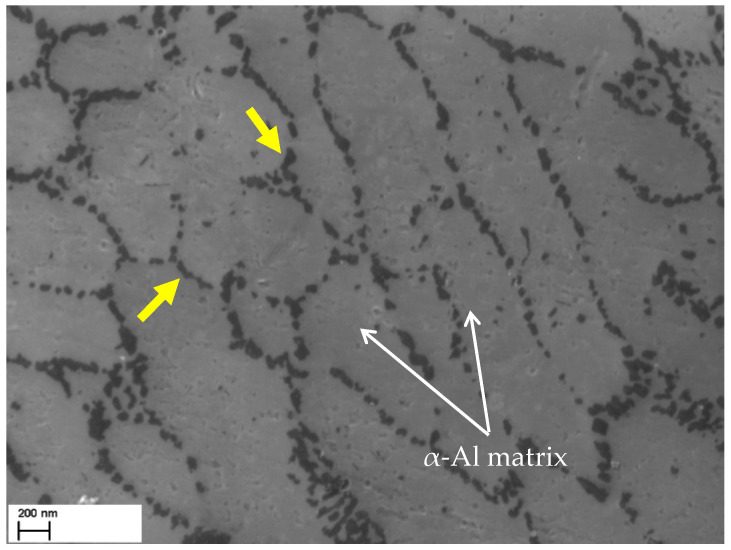
SEM micrograph of an AlSiMg sample in as-built condition. The yellow arrows indicate the Si-eutectic particles that form the Si-network.

The fine microstructure of the as-built AlSi7Mg samples has already been studied in our previous publications [5,15,16]. It is formed by a fine Si-eutectic network (yellow arrows in Figure 2) that surrounds nano-sized α-Al cells (Figure 2), where the precipitation phenomena related to the GP zone → β-Mg_2_Si sequence take place. This cellular microstructure characterizes the entire MP (molten pool) region, from its center to its boundaries. In the adjacent zones of the MP boundaries, the heat fluxes developed during the powder fusion process induced coarsening phenomena of the Si-eutectic and the consequent Si-network crumbling.

**Figure 3:**
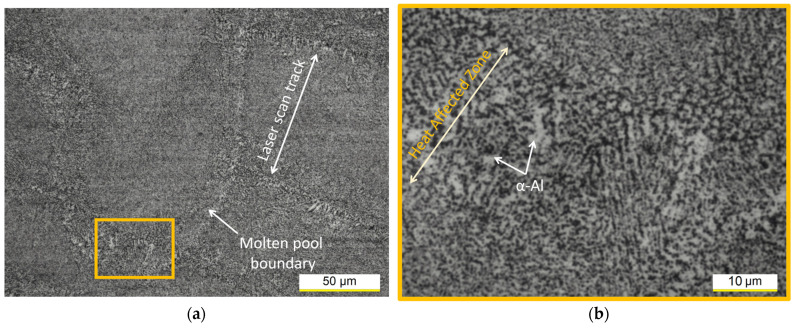
OM micrographs of the bottom AlSi7Mg sample DA at 175 °C per 512 h at 500× (**a**) and 2000× (**b**) magnification acquired on the xy plane. OM micrograph in (**b**) is contained in the yellow frame shown in (**a**).

Figure 3 shows the heterogeneous microstructure of the AlSi7Mg alloy after DA at 175 °C per 512 h. On the xy plane shown in Figure 3a, both the LSTs (laser scan tracks) and MPs, which are arranged according to the scanning strategy used to manufacture these samples (see [16]), highlighted the coarsening phenomena that affect the Si-eutectic network along their boundaries. The magnified microstructure shown in the yellow panel (Figure 3b) highlights a coarsened structure within the heat-affected zone that causes the coarsening phenomena of the α-Al matrix. Other studies [2,9,24,25] have confirmed the same findings. Based on the results discussed by [42], the heat-affected zone is considered the weakest zone of the L-PBF AlSi10Mg samples due to (i) its microstructural features, (ii) the presence of defects, and (iii) the non-uniform stress concentration at the MP boundaries.

**Figure 4:**
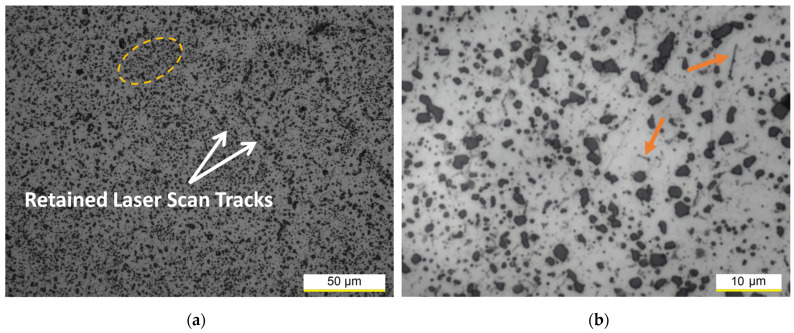
OM micrographs of the AlSi7Mg samples solution heat treated (SHTed) at 505 °C per 0.5 h at 500× (**a**) and 2k× (**b**) magnification. The dotted circle contains the assembled Si-particles and while the orange arrows indicate the Fe-rich phases.

The SHT at 505 °C per 0.5 h transforms the fine α-Al cellular structure in a composite-like microstructure in which the Si-eutectic particles are embedded into the α-Al matrix (Figure 4). The dissolution process of the Si-eutectic network and the consequent decrease of mechanical properties occur after 2.5 min at 505 °C, as already reported in our previous study [16] and by other studies [2,26]. The heterogeneity characterizing the as-built and DA microstructures (Figure 3) can be also observed in Figure 4. The Si-eutectic particles are non-uniformly distributed in the α-Al matrix, showing that they can be assembled (dotted circle in Figure 4a) or more concentrated along the retained LST boundaries (Figure 4a). The highest concentration of Si atoms along the as-built LST boundaries facilitates the formation of coarse Si-eutectic particles, and their subsequent coalescence, along the same LSTs, during the exposure at the SHT temperatures [23]. In the surrounding regions, the α-Al matrix becomes more visible due to the diffusion and subsequent impoverishment of the Si atoms. The orange arrows in Figure 4b indicate the presence of needle-like Fe-rich phases that are coarsened during the 0.5 h soaking time at 505 °C. Several studies [2,43] have confirmed the nature of these intermetallic phases through XRD analysis and EDX maps.

**Figure 5:**
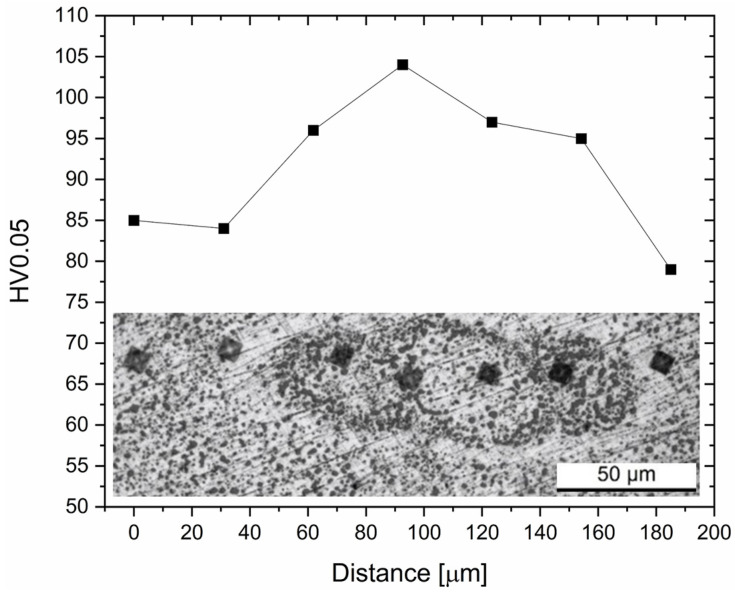
Vickers microhardness profile performed on a residual MP in the AlSi7Mg alloys SHT at 505 °C per 0.5 h. The indentations were carried out with a load of 5 gf and a dwell time of 15 s.

The Si atoms’ diffusion promoted softening effects in the adjacent zones to the LSTs, as indicated by the lowest Vickers microhardness values (80–85 HV0.05) characterizing those regions (Figure 5).

**Figure 6:**
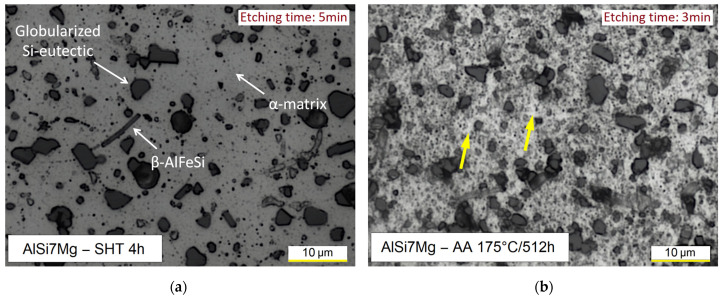
OM micrographs (2k×) of the AlSi7Mg samples SHTed at 505 °C per 4 h: before (**a**) and after (**b**) the AA at 175 °C per 512 h. The OM micrographs were acquired after both 5 (**a**) and 3 min (**b**) of chemical etching in Keller’s reagent. The yellow arrows indicate the PFZ.

When the soaking time at the SHT temperatures was increased, the diffusion kinetics induced coarsening phenomena of both the Si-eutectic particles and needle-like Fe-rich phases (compare Figure 4b with Figure 6a). At the same time, the distribution of the Si-eutectic particles within the α-Al matrix became more homogeneous and the amount of the retained LSTs decreased, as reported in our previous studies [15,16]. The same studies concluded that the fine Si-eutectic particles belonging to the eutectic network increase their dimensions from 0.05 ± 0.01 μm to 4.80 ± 0.60 μm.

The exposure time of 3 min in the Keller’s reagent highlighted the precipitates that formed during the AA at 175 °C within the α-Al matrix (Figure 6b). Considering that they are detectable through OM observations, it is possible to conclude that they were affected by coarsening phenomena. At the same time, the loss of alloying element by the diffusion process promoted the formation of several precipitate-free zones (PFZs), as indicated by the yellow arrows in Figure 6b. Other studies [44,45] have highlighted the same microstructural conditions by evaluating the precipitation phenomena that occurred in cast Al alloys through a chemical etching. Since the PFZs are generally disposed along the grain boundaries, Figure 6b confirms that the nano-sized grains of Al–Si–Mg as-built samples are subjected to coarsening phenomena as also confirmed by [2,5,22]. Other studies [22,28] have reported that the recrystallization process affects the nano-sized grains during exposure to temperatures higher than 520 °C.

### 3.2. Aging Profiles

**Figure 7:**
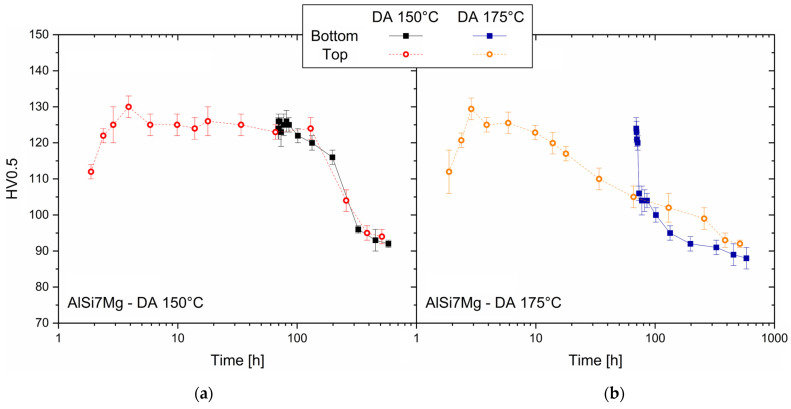
Aging curves related to the DA at 150 °C and 175 °C and carried out on the top (dotted lines) and bottom (continuous lines) regions of the AlSi7Mg bars. The time axis considers both the soaking time onto the pre-heated build platform and the exposure at the DA temperatures: 150 °C (**a**) and 175 °C (**b**).

The strengthening effects induced by the DA on the as-built AlSi7Mg samples were assessed by the aging profiles shown in Figure 7. The aging curve of the bottom (continuous lines) samples moved towards longer times due to their soaking time on the pre-heated build platform. For that reason and the consequent influence on the precipitation phenomena [5], the Vickers microhardness increased from the top regions (first points of all dotted lines, Figure 7) to the bottom ones (first points of all continuous lines, Figure 7).

The DA at 150 °C (Figure 7a) induced a maximum strengthening of +18 ± 1 HV0.5 at 2 h soaking time, and peak aged conditions (130 ± 3 HV0.5 of the top AlSi7Mg samples). For longer time periods (t < 130 h), the holding time at 150 °C promoted a slight softening phenomenon on both the top and bottom samples probably due to the coarsening effects of the semi-coherent β″- and β′-precipitates. By increasing the holding time up to 512 h, the possible β′ → β transformation induced an exponential decrease of the Vickers microhardness to 92 ± 2 HV0.5 and 94 ± 2 HV0.5 for the bottom and top samples, respectively. These Vickers microhardness values seem to represent the asymptotic values of the AlSi7Mg alloys DA at 150 °C. Figure 7b shows the aging profiles, obtained at 175 °C, in which their initial points represent the as-built conditions of both the top and bottom samples after the exposure at 150 °C (build platform temperature). The aging profile of the top AlSi7Mg sample in Figure 7b shows a peak aging condition (129 ± 3 HV0.5 at 1 h of exposure at 175 °C). For longer time periods, the Vickers microhardness values monotonically decreased to 92 ± 2 HV0.5. The bottom samples, however, described a severe decrement of the Vickers microhardness during the first hours (*t* < 4 h) of the exposure at 175 °C. On the contrary, the Vickers microhardness values reached 88 ± 3 HV0.5 at 512 h by describing a less steep trend during exposure times higher than 4 h.

Finally, it is possible to conclude that the long-term DA at both temperatures led both the top and bottom samples of AlSi7Mg (Figure 7) to stability in terms of Vickers microhardness. These conditions were reached more quickly during the exposure at 175 °C (Figure 7b,d) due to the possible coarsening phenomena, as will be discussed later.

**Figure 8:**
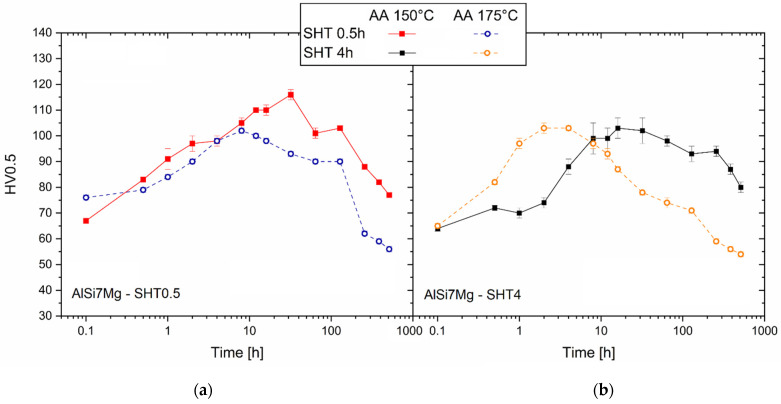
Aging curves related to the AA at 150 °C and 175 °C carried out on the AlSi7Mg samples SHTed per 0.5 (**a**) and 4 h (**b**). Considering that the exposure time is expressed through a logarithmic axis, the SHTed conditions are represented by the time equals to 0.1.

Figure 8 shows the strengthening effects induced by the AA at 150 °C and 175 °C on the AlSi7Mg samples SHTed per 0.5 h (Figure 8a) and 4 h (Figure 8b). As examined in our previous study [16], the SHT promoted relevant reductions in the Vickers microhardness of the as-built AlSi7Mg samples (Table 3) due to the microstructural variations already discussed in Section 3.1.

Focusing on the AlSi7Mg alloys SHTed per 0.5 h (Figure 8a), the highest peak aging values of 116 ± 1 HV0.5 were obtained after an exposure time of 32 h at 150 °C. By increasing the AA at 175 °C, the peak aging was shifted in the lower left, given that the highest Vickers microhardness of 102 ± 1 HV0.5 was reached with 8 h soaking time. The same peak aging values of 103 ± 1 HV0.5 were obtained by aging at 150 °C per 16 h or 175 °C per 2 h and the AlSi7Mg samples SHTed per 4 h (Figure 8b).

For longer exposure times, the AA at 175 °C conferred the highest softening effects, as demonstrated by Vickers microhardness values lower than those characterized in the SHT conditions. This phenomenon is intensified in the samples SHTed per 4 h. On the contrary, the DA at 150 °C induces smaller driving forces related to the softening phenomena that occurred during the analyzed soaking time range (0.5–512 h).

### 3.3. Mechanical Properties

**Figure 9:**
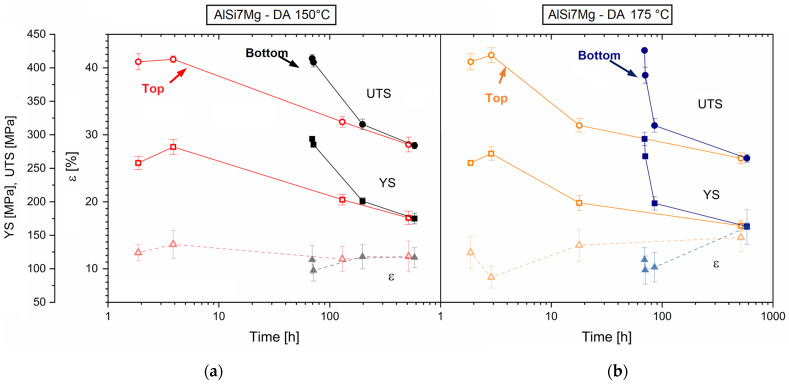
Mechanical properties of AlSi7Mg samples after DA at 150 °C (**a**) and 175 °C (**b**). Top samples are described by red and orange lines and bottom ones by black and blue lines.

Figure 9 shows the mechanical properties variations of AlSi7Mg samples after the DA at 150 °C (Figure 9a) and 175 °C (Figure 9b). Generally, both the UTS and YS follow the same trends of the aging profiles described in Figure 7. At the peak aged conditions, the UTS and YS reached the same values after both the DA at 150 °C (Figure 9a) and 175 °C (Figure 9b), as already shown by the aging profiles (Figure 7). Due to the precipitation phenomena that occurred in the peak aged top samples, the highest increments (+ 9.3% and + 5.4%) were obviously revealed on the YS values, considering that they are deeply influenced by the precipitate-dislocation interactions. In view of the over-aged conditions reached during the long-term exposure at the DA temperatures, the coarsened precipitates led the tensile strength at values lower than those obtained by the as-built samples. For the same reasons, the top samples exhibited comparable strength to the bottom ones (t > 128 h). The unique significant result was obtained after 512 h soaking time at 175 °C (Figure 9b), in which the ductility values increased from about 8% (as-built) to ~15.5%.

**Figure 10:**
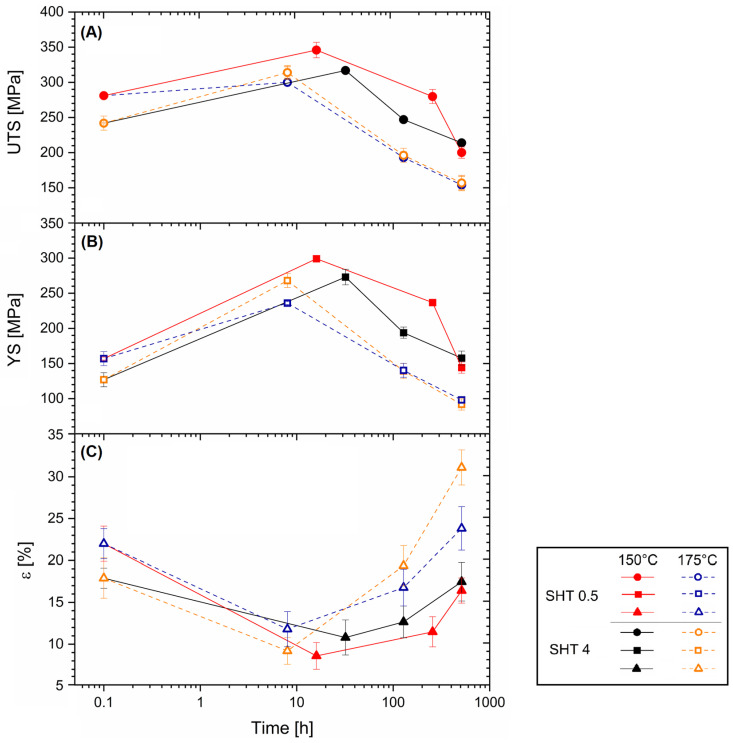
Mechanical properties of AlSi7Mg SHTed per 0.5 and 4 h and DA at 150 °C (continuous lines) and 175 °C (dotted lines): (**A**) UTS, (**B**) YS, and (**C**) elongation values.

Figure 10 shows the mechanical properties variations of the SHTed AlSi7Mg samples after the AA at 150 °C (continuous lines) and 175 °C (dotted lines). The effects induced by the SHT on the as-built microstructure (Figure 4 and Figure 6) were obviously reflected on both the UTS (Figure 10a) and YS (Figure 10b) values. The worst effect (demonstrated by comparing Figure 9 with Figure 10) was exhibited by the samples SHTed per 4 h, which showed UTS and YS values of 241 ± 10 MPa and 127 ± 2 MPa, respectively. The lower degree of the coarsening phenomena that affected the Si-eutectic particles (Figure 4) during the SHT per 0.5 h conferred higher UTS (+16.5%) and YS (+24%) values than those shown by the samples SHTed per 4 h. During the AA at both 150 °C and 175 °C, the UTS and YS values increased up to the maximum (peak aged conditions) and then decreased, as also previously exhibited by the aging curves (Figure 8). Also in this case, the AA at 175 °C conferred the worst effects on both the UTS and YS values due to the greater coarsening phenomena that affected the precipitated phases.

Generally, the AlSi7Mg samples artificially aged at 150 °C, after the SHTed per 0.5 h (red lines), continued to describe the highest peak aged conditions by reaching 317 ± 3 MPa and 299 ± 4 MPa of UTS and YS, respectively. Despite these improved conditions, the DA AlSi7Mg samples in peak aged conditions exhibited higher UTS values, of +18.5% (DA at 150 °C) and + 32% (DA at 175 °C), respectively. On the other hand, the YS values of AA AlSi7Mg reached those obtained in DA conditions (Figure 9a), due to the substantial number of precipitates formed during the artificial aging. Simultaneously, the peak aged elongations (9.2–11.8) percentages are also equal to the ductility shown by the DA AlSi7Mg ((9.8–13.7)%, Figure 9a).

For longer exposure times, the coarsening effects that affected the precipitates (Figure 4) and the consequently reduction in the SSS of the α-Al matrix led the elongations at values comparable to those shown by the Al–Si–Mg samples in SHTed conditions. The highest softening effect was exhibited by the AlSi7Mg samples SHTed per 4 h, where the elongation increased from 18 ± 1% to 31 ± 2%. At the same time, the AA at 175 °C generally confers higher ductility values compared to those obtained after the AA at 150 °C (Figure 9c).

## 4. Discussion

### Aging Effects on Mechanical Properties

**Figure 11:**
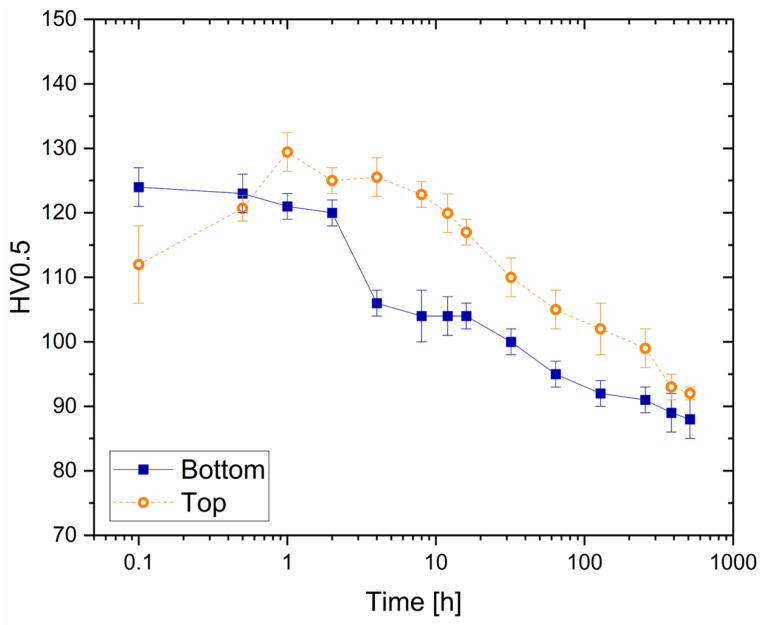
Aging curves of both to the top and bottom AlSi7Mg samples DA at 175 °C. The time axis represents the actual aging time at 175 °C (Table 2).

The presented results confirmed that only the top regions of the as-built AlSi7Mg bars have the potential to respond to the DA at 150 and 175 °C by increasing their mechanical properties. The aging curves showed a Vickers microhardness increment, as well as the strength values, up to the peak aged conditions (dotted lines in Figure 7 and Figure 9) due to the presence of Si and Mg atoms within the lattice structure of the α-Al matrix. Several studies reported in [2] have confirmed the presence of a SSS in Al–Si–Mg samples additively manufactured on a pre-heated build platform. On the other hand, a long residence time on the build platform pre-heated at 150 °C created over-aging conditions in the bottom samples (continuous lines in Figure 7 and Figure 9). The Vickers microhardness and tensile strength decreased with the exposure time at 150 and 175 °C. In the former case, the aging profiles of the bottom samples (Figure 7a,c), which followed the trends described by the top ones, exponentially decreased in the range of 130–256 h soaking time and then settled down to 96 ± 4 HV0.5. In the latter case, the DA at 175 °C can be considered as a double aging that (i) promoted peak aging conditions on both the top samples already aged at 150 °C (dotted lines in Figure 7c,d), and (ii) slightly decreased the Vickers microhardness in the bottom regions during the first times of exposure (Figure 7c enlarged in Figure 11). The Vickers measurements slightly decreased from 125 ± 4 HV0.5 to 120 ± 3 HV0.5 during the first hours of the exposure at 175 °C.

The DA at 175 °C can cause a precipitation phenomenon of the remaining Si and Mg alloying elements in the α-Al matrix of the top AlSi7Mg [4]. As reported in the XRD analysis performed in our previous study [5] and by other authors [4,8,9,24,28], the precipitation phenomena occurring in the top samples formed nano Si-particles and triggered the precipitation sequence of the β-Mg_2_Si. Focusing on the bottom regions, the higher exposure time on the pre-heated build platform increased the quantity of nano Si-particles, due to the diffusion of Si atoms from the SSS in the α-Al matrix, and accelerated the β″ → semi-coherent β′ → β-Mg_2_Si transformation, leading to the bottom over-aging (Figure 7). The following exposure at 175 °C can promote new precipitation phenomena, which can explain the peak aging conditions shown in Figure 7, as widely analyzed by Marceau et al. [46]. The same authors evaluated the effects induced on a double aged Al–Cu–Mg alloy. Other studies [47,48] have confirmed the same findings on a cast Al–Mg–Si–Cu alloy and on a 7050 alloy, respectively. The lower amount of the alloying elements in the bottom than in the top of the α-Al matrix, as well as the greater coarsening effects affecting the formed precipitates in as-built samples, can promote a small amount of new precipitate. Therefore, the precipitation phenomena of new phases slows down the reduction in the Vickers microhardness values.

Focusing on the AA curves obtained at the same aging temperatures described above (Figure 8), it is possible to conclude that the SHTed LPBF alloys become more sensitive to aging temperatures. The Vickers microhardness variations between the starting point and the peak aging were more pronounced than in the as-built cases (Figure 7). The precipitation sequence triggered by the AA at 150 °C produces Vickers microhardness at the highest values in AlSi7Mg SHTed per 0.5 h. These peak aging conditions are, however, higher than those obtained after the SHTed per 4 h, probably due to the lower wt% Mg in the SSS. The holding time at high temperatures reduces the percentage of the alloying elements within the α-Al lattice structure. In other words, it leads to the high Si and Mg concentrations of as-built samples towards the equilibrium conditions [2,28]. Tonelli et al. [4], who analyzed the effects of T6 heat treatment on the L-PBF AlSi7Mg0.6 alloy, affirmed that precipitation of Si atoms had already occurred after 10 min at SHT temperatures. Increasing the AA temperature to 175 °C, both the AlSi7Mg samples SHTed per 0.5 h showed lower peak aging values that were reached for shorter exposure time (Figure 8a,c). An abnormal behavior was described in this study by the same alloys SHTed per 4 h (Figure 8b and Figure 10); the Vickers microhardness, as well as the UTS and YS values, at the peak aging, match those obtained after both the SHTed per 4 h + AA at 150 °C (Figure 8a and Figure 10) and the SHTed per 0.5 h + AA at 175 °C (Figure 8b and Figure 10). Through the different β-Mg_2_Si morphology and size obtained by solution heat treating a cast A356 alloy with different soaking times, Asghar et al. [49] produced the same conditions detected in the present work. The same authors observed smaller needle-shaped β″ precipitates in 8 h solution-treated peak aged (AA at 180 °C) than in 6h ones. Future studies will probably be focused on further microstructural analysis of these aspects. The different soaking times at 505 °C also influenced the mechanical performance in long-term aging. Although the trends of the aging curves obtained at 150 and 175 °C are very similar (Figure 8a,c), those obtained for alloys SHTed per 4 h lead to the conclusion that AA at 150 °C and 175 °C followed different precipitation paths (confirming the statements previously discussed regarding the peak aged samples). The coarsened precipitates formed at 512 h soaking time (Figure 4c,d) explain the exponential decreases of the Vickers microhardness that characterized the peak aged conditions. More specifically, the Vickers microhardness values were smaller than those measured in the SHT conditions after 128 h of soaking time.

**Figure 12:**
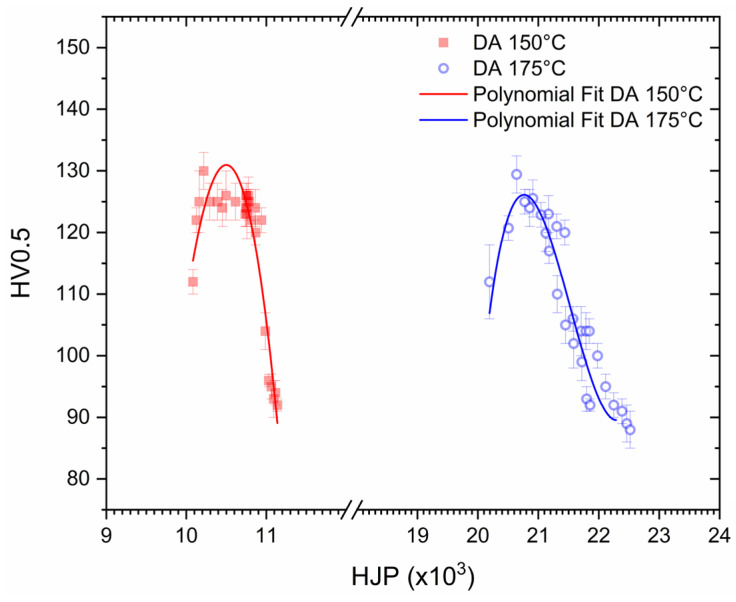
Hollomon–Jaffe Parameter versus Vickers microhardness measured on AlSi7Mg samples DA at both 150 (red symbols) and 175 °C (blue symbols). The continuous lines represent the polynomial fits at second order.

**Figure 13:**
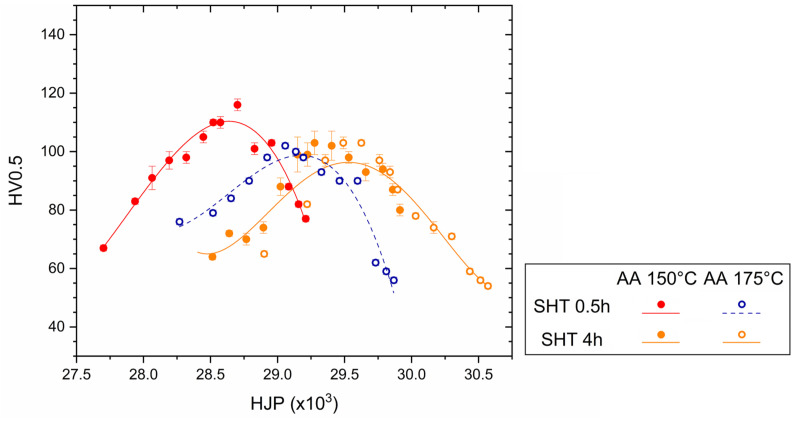
Hollomon-Jaffe parameter versus Vickers microhardness measured on AlSi7Mg SHTed at 505 °C per 0.5 (red and blue symbols) and 4 h (orange symbols) and then AA at both 150 (red and orange symbols) and 175 °C (blue and orange symbols). Both the continuous and dotted lines represent the polynomial fits.

To compare the responses obtained by the heat treatments (Table 2), the measured Vickers microhardness values were correlated to the temperature (T, [K]) and time (t, [s]) variables of both the DA (Figure 12) and AA (Figure 13) through the following Hollomon–Jaffe parameter (HJP):

(1) $$HJP=T\left(k+\log t\right)$$

where k is a constant equal to 20 for the aluminium alloys [50,51]. The exposure times at the build platform temperature and the SHT conditions were considered in the evaluation of the HJPs of both the DA and AA conditions. Focusing on DA AlSi7Mg, Figure 12 exhibits two different zones: (i) a region with an increasing trend up to the peak aging conditions, followed by (ii) a region in which the over aging conditions induced an exponentially decreasing trend. The AlSi7Mg samples DA at 175 °C showed lower peak aged conditions. Considering the HJPs higher than 20 × 10^3^, the softening phenomena are more pronounced in the AlSi7Mg samples.

In relation to the AA effects on the AlSi7Mg samples, Figure 13 shows the same two different regions described in Figure 12. Due to the trends of the profiles discussed in Figure 7a, the HJPs continue to show a variance between the AlSi7Mg DA at 150 °C (dotted lines) and 175 °C (continuous lines) On the contrary, the SHT per 4 h seems to equate the effects obtained after both the AA AlSi7Mg alloys (orange symbols).

## 5. Conclusions

Long-term effects of several direct and artificial aging heat treatments were studied on laser powder bed-fused AlSi7Mg. Based on the results presented, the following conclusions can be drawn:

1. The α-Al cells and the Si-eutectic network dissolved during the SHT per 0.5 h, while coarsening phenomena of the Si-eutectic particles and Fe-rich phase affected the AlSi7Mg SHTed per 4 h. Despite these microstructural variations, the retained laser scan tracks continued to induce a certain degree of microstructural heterogeneity.
2. Detrimental effects on the Vickers microhardness and tensile strength were obtained after 128 h exposure time at 150 °C, both in as-built and SHTed samples.
3. DA at 175 °C induced secondary aging effects on both the top and bottom samples and promoted a faster decrease of the mechanical properties. As regards the samples SHTed for the longest soaking times, the AA at 175 °C led to mechanical properties at values lower than those exhibited by the samples in SHTed conditions, due to the presence of coarsened precipitates.
4. The more homogenous microstructure that characterized the samples SHTed per 4 h allowed the best improvement to be obtained in terms of ductility after the AA at 175 °C per 512 h (increment of + 35% with respect to the SHTed conditions).
5. The AlSi7Mg samples that are characterized by the cellular α-Al matrix seem to endure better long-term exposures at both 150 °C and 175 °C, probably because the fine and cellular microstructure continues to provide adequate stiffness and strength despite the coarsening phenomena affecting the precipitates.

## Figures and Tables

**Table 1:** Chemical composition in wt% of the gas-atomized AlSi7Mg powders.

Alloys	Si	Mg	Fe	Cu	Mn	Ti	C	Al
AlSi7Mg	7.0	0.6	0.06	<0.005	0.006	0.12	0.03	Balance

**Table 2:** Heat treatment conditions performed on both the AlSi7Mg bars and tensile samples.

Heat Treatments	Temperature [°C]	Time [h]
DA and AA	150	0.5, 1, 2, 4, 8, 12, 16, 32, 64, 128, 256, 384, 512
	175	
SHT	505	0.5
		4

**Table 3:** Vickers microhardness of SHTed AlSi7Mg bars [16].

Alloys	SHTed per 0.5 h		SHTed per 4 h	
	HV0.5	ΔHV0.5 ^1^	HV0.5	ΔHV0.5 ^1^
AlSi7Mg	75 ± 1	−36%	62 ± 1	−48%

^1^ Percentage calculated with respect to the as-built Vickers microhardness values.

## Data Availability

Data are contained within the article.
